# Transition from poor ductility to room-temperature superplasticity in a nanostructured aluminum alloy

**DOI:** 10.1038/s41598-018-25140-1

**Published:** 2018-04-30

**Authors:** Kaveh Edalati, Zenji Horita, Ruslan Z. Valiev

**Affiliations:** 1 WPI, International Institute for Carbon-Neutral Energy Research (WPI-I2CNER), Kyushu University, Fukuoka, 819-0395 Japan; 2 Department of Materials Science and Engineering, Faculty of Engineering, Kyushu University, Fukuoka, 819-0395 Japan; 3 Institute of Physics of Advanced Materials, Ufa State Aviation Technical University, Ufa, Russia; 4 Laboratory for Mechanics of Bulk Nanomaterials, Saint Petersburg State University, Saint Petersburg, Russia

## Abstract

Recent developments of nanostructured materials with grain sizes in the nanometer to submicrometer range have provided ground for numerous functional properties and new applications. However, in terms of mechanical properties, bulk nanostructured materials typically show poor ductility despite their high strength, which limits their use for structural applications. The present article shows that the poor ductility of nanostructured alloys can be changed to room-temperature superplastisity by a transition in the deformation mechanism from dislocation activity to grain-boundary sliding. We report the first observation of room-temperature superplasticity (over 400% tensile elongations) in a nanostructured Al alloy by enhanced grain-boundary sliding. The room-temperature grain-boundary sliding and superplasticity was realized by engineering the Zn segregation along the Al/Al boundaries through severe plastic deformation. This work introduces a new boundary-based strategy to improve the mechanical properties of nanostructured materials for structural applications, where high deformability is a requirement.

## Introduction

Nanostructured materials with ultrafine grains (UFG) in the range of submicrometer or nanometer have been of high interest for the last decades due to their unique atomic structure and unusual properties for advanced functional applications^1^. Nanostructured metallic materials in the bulk form, which are usually produced by consolidation of nanopowders^2^ or by severe plastic deformation (SPD) of bulk samples^3^, are considered as candidates for structural applications because of their superior strength^4^. However, these materials typically exhibit poor plasticity and ductility, which limits their use for many structural applications^5^.

Plasticity of nanostructured materials at a temperature of *T* strongly depends on the homologous temperature, *T*/*T*_*m*_ (*T*_*m*_: melting point). At homologous temperatures smaller than ~0.5, deformation is mainly controlled by the mechanisms based on dislocations motion^6^. Since the dislocation activity becomes limited within the small nanograins, the plasticity of nanostructured materials is typically poor at low homologous temperatures^5^. However, at homologous temperatures higher than ~0.5, thermally-activated deformation mechanisms such as grain-boundary sliding or coble creep become dominant^5,6^. As a result of the transition in deformation mechanism and significant contribution of grain boundaries to deformation, the nanostructured materials can show high plasticity and even superplasticity (i.e. elongations over 400% under tension) at high homologous temperatures despite their limited plasticity at room temperature^7^. Therefore, the room-temperature plasticity of nanostructured materials could be improved and even reach the superplastic range (i.e. over 400%), if the dominant deformation mechanism at room temperature can be tuned to the grain-boundary sliding. Such high plasticity values cannot be achieved using the strategies developed earlier for the improvement of plasticity (mainly uniform ductility) of nanostructured materials based on the dislocation activity^8^, twinning^9^ or phase trsnsformation^10^.

Grain-boundary sliding can be usually described by the following strain rate - stress ($\dot{\varepsilon }-\sigma$) relationship for a material with a shear modulus of *G*, a Burgers vector of *b* and an average grain size of *d*^11^.

1 $$\varepsilon =\frac{AD\dot{G}b}{kT}{(\frac{b}{d})}^{p}{(\frac{\sigma }{G})}^{n}$$

In this equation, *A* is ~10^7^, *k* is Boltzmann’s constant, *p* is inverse grain size exponent, *n* = 1 /*m* is stress exponent (*m*: strain-rate sensitivity which is defined as the slope of $\dot{\varepsilon }-\sigma$ in a double-logarithmic plot) and $D={D}_{0}\exp (\,-\,Q/RT)$ is the diffusion coefficient along grain boundaries (*D*_0_: pre-exponential frequency factor, i.e. maximal diffusion coefficient at infinite temperature, *Q*: activation energy and *R*: gas constant)^12^. On the basis of equation (1), grain size reduction has been realized as an effective strategy for domination of grain-boundary sliding at low temperatures and achieving superplasticity^13^. Although earlier studies revealed that the temperature for superplasticity might be reduced by 100–200 K by reducing the grain size to the nanometer range^13^, the temperatures for superplastic deformation are typically higher than 0.5*T*_*m*_ (e.g., 473 K, 523 K and 923 K for Mg-based, Al-based and Ti-based alloys^14^) except for a Mg-Li alloy^15^, and only the alloys with low-melting temperatures (e.g., Sn-based^16^, Pb-based^17^ and Zn-based^18^ alloys) can exhibit room-temperature superplasticity. A new strategy for domination of grain-boundary sliding that can be realized from equation (1) is enhancement of diffusion by engineering the grain-boundary structure and segregation.

In this study, we show that engineering of segregation of Zn atoms in an Al-Zn alloy via SPD would enhance the diffusion and grain-boundary sliding and lead to the occurrence of room-temperature superplasticity. The alloy demonstrated high tensile plasticity over 400% at a homologous temperature of 0.36 *T*_*m*_ for the first time in any Al-based alloys. The Al-Zn system was selected because earlier studies using first-principles calculations confirmed that the segregation of Zn atoms along Al/Al grain boundaries can enhance the grain-boundary energy and diffusion^19^. The SPD method was selected for material processing because earlier experiments showed that the method allows for not only producing ultrafine grains but also enhancing the Zn segregation in the Al/Al boundaries^20^. The current study presents a new grain-boundary-based strategy to improve the room-temperature plasticity of nanostructured alloys for structural application.

## Materials and Methods

The initial alloy was fabricated by addition of 30 at.% of Zn to Al by ingot melting. The alloy had liquidus and solidus temperatures of 830 K and 750 K, respectively, and contained two immiscible Al-rich α and Zn-rich η phases, as shown in the phase diagram of Fig. S1(a)^21^. The Zn concentration was intentionally chosen high to make sure that all boundaries are covered by Zn segregation after SPD processing. The ingots, which had 30 × 20 × 80 mm^3^ dimensions, were homogenized at 703 K for 24 h and subsequently cooled down in air. To achieve the grain-boundary segregation by SPD processing, the homogenized ingot was cut into discs with 0.8 mm thickness and 10 mm diameter and subsequently processed by the high-pressure torsion (HPT) technique^3,22^ (the principles of this SPD method is shown in Fig. S1(b)). The discs were placed between the holes of two HPT anvils (10 mm diameter and 0.25 mm depth), and processed at room temperature under a pressure of 6 GPa with rotating the lower anvil with respect to the upper anvil for *N* = 200 cycles with 1 rpm rotation speed. Such a large number of SPD cycles was intentionally chosen to maximize the segregation of Zn atoms because recent studies clearly showed that the distribution of elements in the immiscible systems is significantly improved after very large number of SPD cycles^23^, although the grain sizes may be saturated quickly only after few cycles^24^. It should be noted that earlier attempts to achieve superplasticity in this alloy after SPD for 5 or 25 cycles were not successful, although the plasticity was in the range of 60%^25^ to 160%^26^. Since first-principles calculations predicted that the Zn segregation at grain boundaries would increase the grain-boundary energy^19^, the stability of grain boundaries after SPD was examined by storage of the disc samples at room temperature for up to 100 days (i.e. after natural aging).

The mechanical properties of the samples after homogenization, SPD and natural aging for 100 days were examined by performing (i) microhardness test using the Vickers method along the disc radii using a load of 50 gf for 15 s; and (ii) tensile tests under strain rates of 5.5 × 10^−5−^10^−2^ s^−1^ at a temperature of 300 ± 2 K (i.e., room temperature during the tests in Fukuoka) using miniature tensile specimens with gauge dimensions of 0.7 × 0.5 × 1.5 mm^3^ (Fig. S1(c) shows the procedure used for cutting the tensile samples from the discs, as described earlier in ref.^15^).

The structural and microstructural features of samples were examined in details at 2–2.5 mm away from the center of discs using (i) X-ray diffraction (XRD) using the Cu Kα source; (ii) atomic force microscopy (AFM) before and after tensile test; (iii) laser microscopy before and after tensile test; (iv) scanning electron microscopy under 15 kV in the backscatter electron (SEM-BSE) mode; (v) Cs-corrected scanning transmission electron microscopy (STEM) under 200 kV in the bright-field (BF), high-angle annular dark-field (HAADF) and energy-dispersive X-ray spectroscopy (EDS) modes using a convergence angle of 46 mrad and detecting angles of 90–370 mrad. The samples for STEM were prepared using the focused ion beam method.

## Results

**Figure 1:**
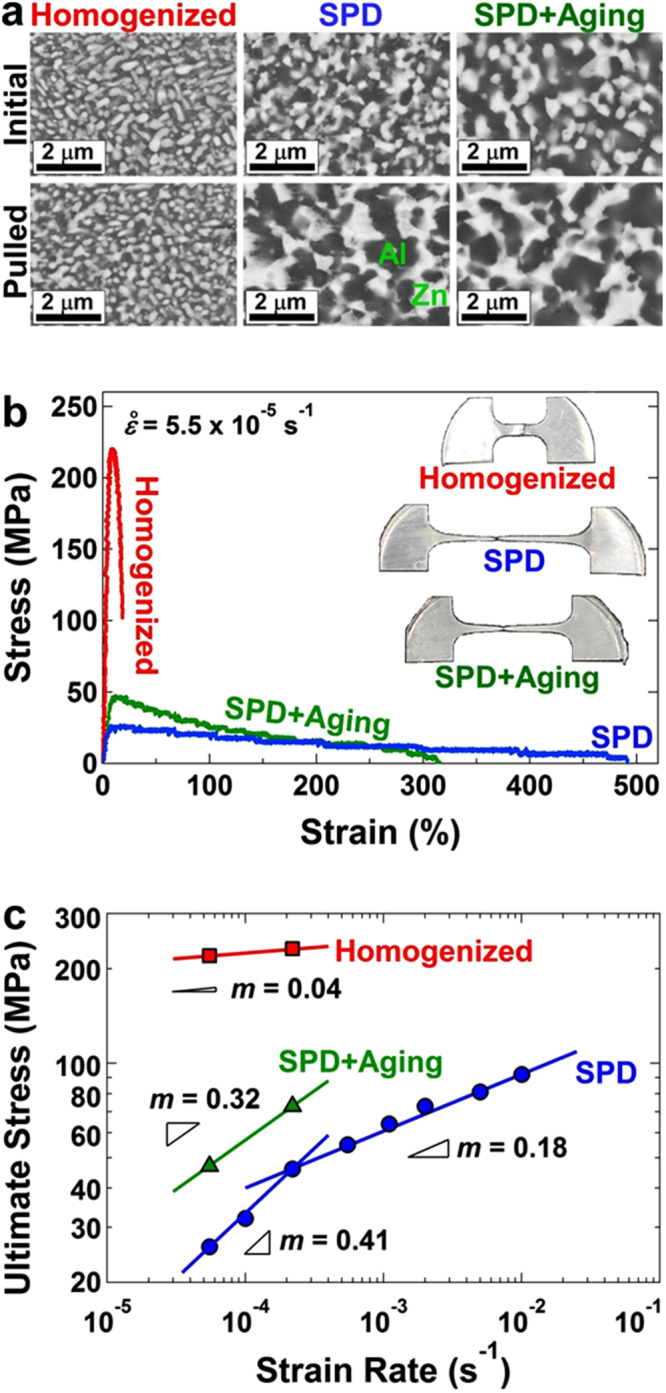
Room-temperature superplasticity with a maximum elongation of 480% and a strain-rate sensitivity of 0.41 is achieved after SPD processing. Microstructure and tensile properties of Al-Zn alloy processed with homogenization, SPD and SPD followed by natural aging for 100 days. (**a**) SEM-BSE images showing the size and distribution of Al- and Zn-rich phases before and after tensile test. (**b**) Stress-strain curves obtained under tension with a strain rate of 5.5 × 10^−5^ s^−1^. Inset: appearance of tensile specimens after pulling to failure. (**c**) Ultimate tensile stress plotted versus strain rate in double logarithmic scale; the slopes show the strain-rate sensitivity.

To examine the structural and microstructural features of three Al-Zn samples, XRD analysis and electron microscopy were conducted. The alloy after homogenization had a dual-phase structure containing an Al-rich phase with the face-centered cubic (fcc) structure and a lattice parameter of 0.40465 nm and a Zn-rich phase with the hexagonal-close packed (hcp) structure and the lattice parameters of *a* = 0.26649 nm and *c* = 0.49468 nm, as shown in XRD profiles of Fig. S2. The lattice parameters of Zn-rich phase remained unchanged but the lattice parameter of Al-rich phase increased after SPD and even more significantly after aging, indicating that the fraction of Zn atoms in the Al-rich phase was reduced (3.4 at.%, 0.7 at.% and 0.1 at.% of Zn after homogenization, SPD and aging, respectively by considering the relationship between the Zn fraction and lattice parameter^27^). Examinations of the three samples by SEM-BSE as shown in Fig. 1(a), and their analysis using the linear intercept method showed that the average sizes of grains were 210 nm, 280 nm and 390 nm after homogenization, SPD and aging, respectively. Examination of microstructures by STEM also confirmed similar results, as shown in Fig. S3. It should be noted that unlike SPD processing, in which the grain refinement is achieved by straining, the significant grain refinement after homogenization is due to the spinodal decomposition^28^. The current results suggest that the three samples have reasonably similar crystal structures with similar grain sizes at the submicrometer range. The slight increase in the grain size after natural aging indicates that the grain boundaries generated by SPD should have high energy. Although the enhanced grain-boundary energy reduces the stability of grain boundaries^29^, it is favorable for the occurrence of grain-boundary sliding^30^.

To examine the plasticity and strain-rate sensitivity of the three samples, tensile tests were conducted at 300 K under different strain rates in the range of 5.5 × 10^−5^–10^−2^ s^−1^. The stress-strain curves generated at 5.5 × 10^−5^ s^−1^ including the appearance of tensile specimens after failure are shown in Fig. 1(b). The maximum tensile stress is plotted versus the strain rate in Fig. 1(c). Although the three samples had reasonably similar two-phase structures and grain sizes, they showed totally different mechanical properties. For example, the homogenized sample showed poor plasticity with a low strain-rate sensitivity of *m* = 0.04, but the sample after SPD became softer and exhibited superplasticity with a maximum elongation of 480% and the strain-rate sensitivity of 0.41 and 0.18 in the range of 5.5 × 10^−5^–2.2 × 10^−4^ s^−1^ and 2.2 × 10^−5^−10^−2^ s^−1^, respectively. The sample after aging showed hardening and a decrease in the plasticity and strain-rate sensitivity. Hardness measurements, as shown in Fig. S4, also confirmed softening by SPD and hardening by aging, in good agreement with the stress-strain curves. Earlier studies also reported SPD-induced softening in the Al-Zn alloys^25,26^. A comparison between the current mechanical property data and the earlier reports on superplasticity and creep^6,7^ suggests that the deformation mechanism shifted from the dislocation activity to the grain-boundary sliding after SPD at strain rates smaller than 2.2 × 10^–4^ s^−1^, but the contribution of grain-boundary sliding was somehow weakened after subsequent natural aging. It should be noted although the two-phase Al-Zn alloys have been known as superplastic materials for several decades^31^; Fig. 1(b) shows the first successful attempt to achieve room-temperature superplasticity in these alloys.

To get further insight into the mechanisms of plastic deformation during tensile test, the microstructures of three tensile specimens in Fig. 1(b) were examined by SEM-BSE after pulling to failure. The examination of microstructures, as shown in Fig. 1(a), showed that while the grain sizes in the homogenized sample remained unchanged after the tensile test, the grain sizes were increased to 550 nm and 500 nm after the tensile test for the samples processed by SPD and aging, respectively. Such strain-induced increases in the size of equiaxed grains after tensile test typically occurs during the superplastic deformation by grain-boundary sliding, when the grain boundaries are in high-energy states^32^.

**Figure 2:**
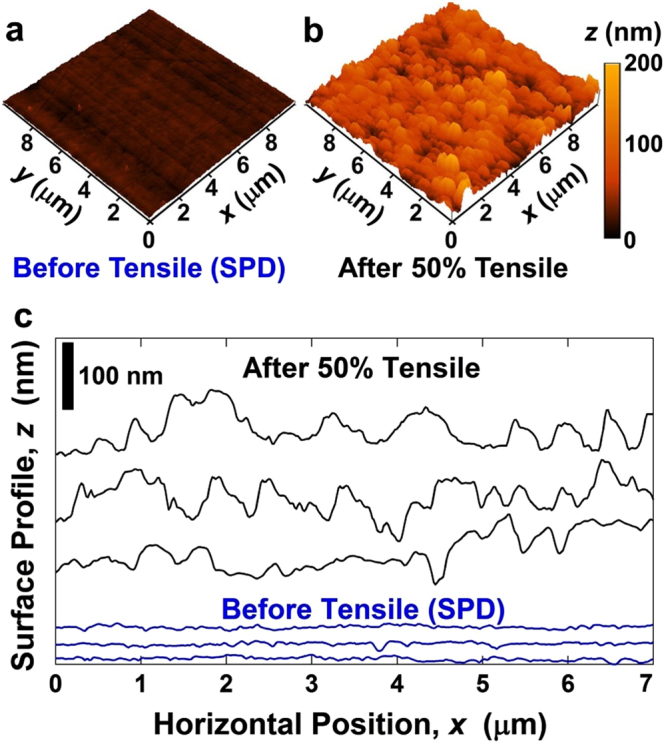
The surface of tensile sample becomes rougher and the average step size perpendicular to the surface increases by ~60 nm after pulling the sample for 50%, indicating the occurrence of grain-boundary sliding. Surface profile before and after tensile test for 50% elongation under a strain rate of 5.5 × 10^−5^ s^−1^ for the sample processed by SPD. (**a**,**b**) AFM images showing the surface roughness. (**c**,**d**) Plots of surface profiles on three different areas of sample achieved by AFM line scanning.

To examine the contribution of grain-boundary sliding to the large plasticity achieved after SPD, a tensile specimen was polished smoothly and pulled by 50% under 5.5 × 10^−5^ s^−1^ at 300 K. The surface of sample was examined before and after tensile test by AFM as shown in Fig. 2(a,b), and the contribution of grain-boundary sliding was determined based on the grain size and the average step size perpendicular to the surface (*v*) as ${\varepsilon }_{GBS}=1.4v/d.$^14^ Since the average value of *v* (measured by AFM over 300 μm^2^ area) increased by ~60 nm (see Fig. 2(c) for some representative data achieved from different areas of sample), the contribution of grain-boundary sliding to the total deformation was estimated ~60%. The value of 60% should be considered as a rough estimation, because the examination of grain boundary sliding by surface roughness measurements do not consider the effect of free surface and assumes that the roughness is caused only by grain-boundary sliding. However, such a high value is not surprising because a recent crystal plasticity model predicted 30% contribution for grain boundary sliding even in a non-superplastic alloy^33^. The examination of surface by laser microscopy, which has lower resolution than AFM at the nanometer range, also showed that the surface became rougher after tensile test, as shown in Fig. S5 for different areas of the sample. These surface analyses clearly confirm that the grain-boundary sliding should be the dominant deformation mechanism at room temperature after SPD which could lead to the softening and significant enhancement of plasticity.

**Figure 3:**
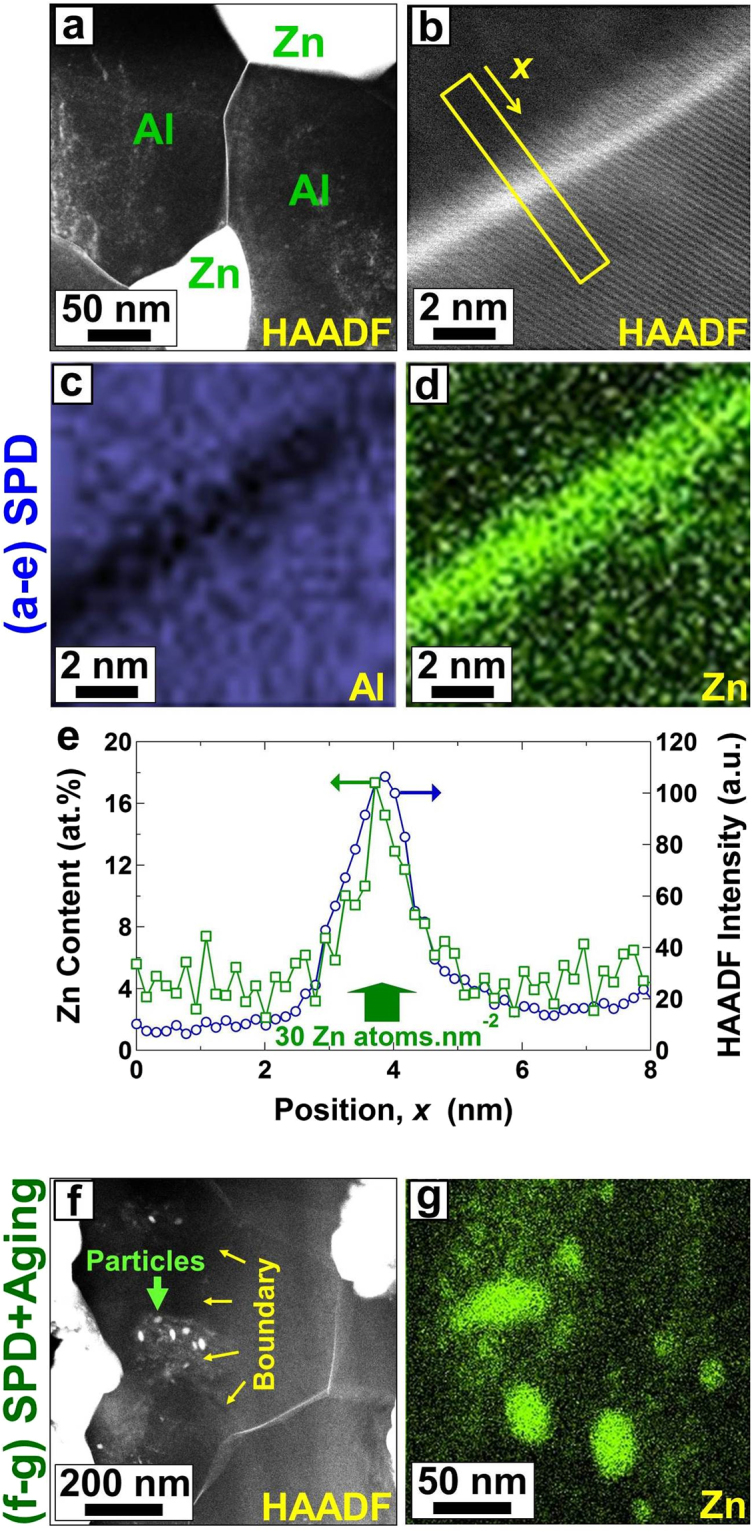
Zn segregates along the Al/Al boundaries after SPD processing, but the Zn-rich boundaries partly transform to Zn precipitates after natural aging. Microstructure of the sample processed by SPD and SPD followed by natural aging. (**a**,**b**) HAADF images showing the distribution of Al and Zn atoms after SPD processing. Bright and dark contrasts correspond to the Al- and Zn-rich areas, respectively. (**c**,**d**) EDS mapping for Al and Zn obtained from the HAADF image of (**b**). (**e**) HAADF and EDS line profiles along the direction indicated by an arrow in HAADF image of (**b**). (**f**,**g**) HAADF image and corresponding EDS mapping for Zn after SPD processing followed by natural aging for 100 days.

To understand the reason for the grain-boundary sliding and superplasticity at room temperature, the grain boundaries were examined carefully using STEM. Inspection of grain boundaries after SPD using STEM-HAADF images (see Fig. 3(a) in which the dark and bright contrasts correspond to the presence of Al-rich and Zn-rich regions, respectively) confirmed the segregation of Zn atoms along the Al/Al grain boundaries. The segregation of Zn atoms was further confirmed at higher resolution using STEM-HAADF and corresponding EDS analysis, as shown in Fig. 3(c,d). Figure 3(e) shows the HAADF and EDS line profiles along the direction indicated by an arrow in Fig. 3(b), confirming the significant enrichment of Al/Al grain boundaries compared with the interior of Al grains. Although the EDS profiles suggest that the Zn concentration at the grain boundary is ~14% higher than the Zn concentration in the interior of grains, the real boundary enrichment should be higher because of the effects of electron beam spread as well as inclination of grain boundary with respect to the electron beam^34^. If the boundary enrichment (i.e. number of segregated atoms per grain boundary area) in EDS line profile of Fig. 3(e) is roughly calculated as ${S}_{GB}=4w\sum _{i=x1}^{x2}(\frac{{c}_{i}}{{a}^{3}})$ (*w*: EDS scanning step which is 0.155 nm, *x*_1_: apparent start position for segregation which is 2.9 nm, *x*_2_: apparent end position for segregation which is 5.3 nm, *C*_*i*_: Zn concentration in at.% in each scanning step of Fig. 3(d), *a*: lattice parameter of Al-rich phase which is 0.40488 nm), the boundary enrichment will be 30 Zn atoms per nm^2^ of grain boundary. Since there are 14 atoms per nm^2^ of the densest atomic plane of Al (i.e. (111) plane of the fcc structure), it can be concluded that the Al/Al boundaries were covered by ~2.1 atomic layers of Zn atoms. Such a large concentration of Zn atoms in the Al/Al boundaries is high enough to accelerate the grain-boundary energy and diffusion^29^ and enhance the grain-boundary sliding based on equation (1). It should be noted that the Zn segregation was visible after natural aging in most of the boundaries, but the main difference after aging was the disappearance of Zn segregation in some boundaries (with probably symmetric structure^19^) and formation of Zn precipitates, as shown in Fig. 3(f). The presence of Zn precipitates is shown more clearly in a higher magnification by EDS analysis in Fig. 3(g) (see Fig. S6 for the complete set of STEM-HAADF and EDS analyses corresponding to Fig. 3(g)). The current grain-boundary analyses confirm that the Zn segregation should be the main origin for the grain-boundary sliding and room-temperature superplasticity.

## Discussion

**Figure 4:**
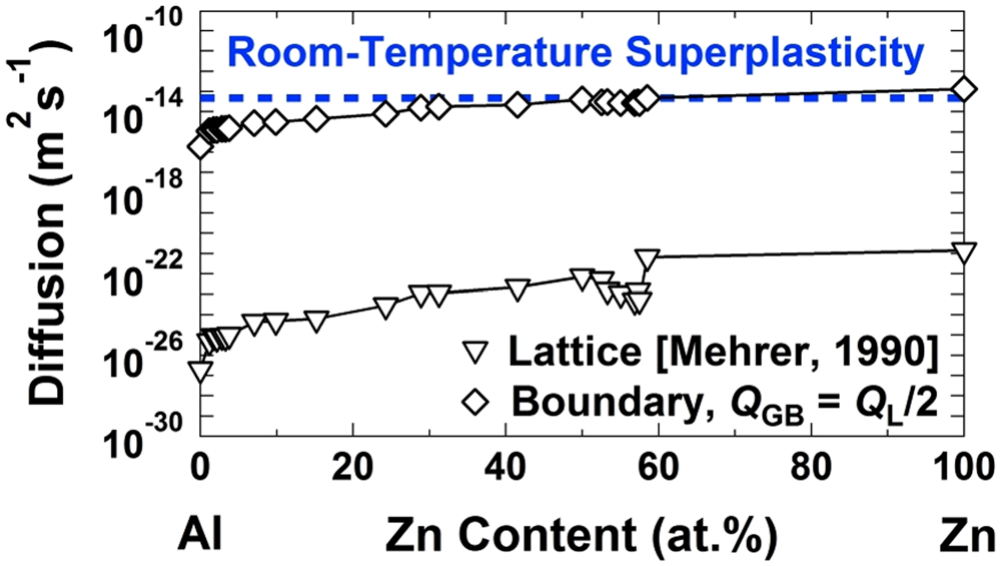
Diffusion coefficient for the room-temperature superplasticity after SPD processing is comparable to the reported data for grain-boundary diffusion in Zn. Lattice and grain-boundary diffusion in the Al-Zn system reported in ref.^12^ in comparison with the diffusion coefficient calculated using equation (1) using the parameters described in the main text for room-temperature superplasticity in this study.

The current results indicate that although two-phase structure is essential to achieve superplasticity in the Al-Zn alloys, room-temperature superplasticity becomes feasible by enhancement of grain boundary segregation through the SPD process. To get further insight into the importance of Zn segregation on enhancement of grain-boundary diffusion and resultant room-temperature superplasticity, the diffusion coefficient can be calculated from equation (1) and compared with the reference data on the diffusivity in the Al-Zn system^12^. Figure 4 shows the reference data for lattice diffusion and grain-boundary diffusion, in which the activation energy for grain-boundary diffusion was considered 50% of the activation energy for lattice diffusion, *Q*_*GB*_ = 1/2 *Q*_*L*_^12^. Using Equation (1) for the superplastic data after SPD (i.e. *σ* = 22 MPa, 32 MPa and 46 MPa at έ = 5.5 × 10^−5^ s^−1^, 1 × 10^−4^ s^−1^ and 2.2 × 10^−4^ s^−1^, respectively) and considering *G* = 26 GPa^6^, $b=a\sqrt{2}/2$  = 0.286 nm, *T* = 300 K, *d* = 280 nm, *p* = 2^7^ and *n* = 1 /*m* = 2.4, the diffusion coefficient for room-temperature superplasticity was determined *D* = (4.6–5.2) × 10^−14^ m^2^s^−1^. This value is two orders of magnitude larger than the diffusivity along the Al/Al grain boundaries and comparable to the grain-boundary diffusion in pure Zn. It should be noted that the Zn/Zn and Al/Zn boundaries are Zn-rich and can exhibit grain-boundary sliding at room temperature. However, based on the linear intercept analysis, 60% of boundaries in the SPD-processed Al-Zn alloy were the Al/Al boundaries, and thus the sliding of these Al/Al boundaries would control the plasticity of the material. As shown earlier in Fig. 3, these Al/Al boundaries were covered by a few layers of Zn atoms during SPD and as a result the grain-boundary diffusion was enhanced in these boundaries and became comparable to the grain-boundary diffusion in Zn. It should be noted that since the Zn atoms have a high driving force to segregate in the boundaries with asymmetric structure (and not with the symmetric structure)^19^, the partial segregation of Zn can be observed even after casting^35^. However, the Zn segregation in the Al-Zn alloys with different compositions (0.35–30% Zn) is accelerated by SPD processing^20,26^ or even by irradiation^36^ due to enhanced atomic diffusion and fast transport of Zn atoms.

Taken together, our study presents a successful example on achieving the room-temperature superplasticity in nanostructured alloys by tuning the deformation mechanism from dislocation activity to grain-boundary sliding. The transition to grain-boundary sliding at a low homologous temperature of 0.36*T*_*m*_ cannot be achieved only by nanograins formation because of three reasons. First, the three samples studied in this paper had reasonably similar two-phase structures and grain sizes, but only one of them exhibited superplasticity. Second, as was shown earlier, the nanostructured Al-based alloys processed by different techniques^14^ and specifically by SPD^37^ cannot exhibit superplasticity below 523 K (i.e. below 0.5*T*_*m*_). Third, earlier works on nanostructured Al-Zn alloys with compositions similar to the one used in this study and processed by SPD for 5 or 25 cycles did not report the achievement of room-temperature superplasticity despite the formation of small grain sizes as 50–380 nm^26^ and ~190 nm^25^. This transition in the deformation should be due to the segregation and enhancement of grain-boundary diffusion. Although earlier studies showed that the segregation along the grain boundaries has a significant effect on the stability and mobility of grain boundaries in alloys^19,20,29^ and ceramics^38^, the current study successfully employed the grain-boundary segregation to achieve the room-temperature superplasticity at such a low homologous temperature for the first time.

The transition from dislocation activity to grain boundary sliding at room temperature can be employed as an effective boundary-based strategy to improve the plasticity of nanostructured materials. This boundary-based strategy which can lead to total tensile plasticity values larger than 400% contrasts with the earlier strategies employed to enhance the uniform plasticity (usually up to a few tenth of percent) by tuning the dislocation activity^8^, twinning^9^ or phase transformation^10^. Since grain-boundary segregation is achievable in many metallic materials by different techniques^39^ including wide range of SPD methods^3,4^, the current study introduces an effective boundary-based strategy to improve the weak plasticity of nanostructured materials.

Finally, it should be noted that the occurrence of superplasticity at room temperature naturally reduces the flow stress which is not desirable for many structural applications. However, room-temperature superplasticity provides a unique opportunity to shape the nanostructured materials to their final shape easily at room temperature. The flow stress of the shaped specimens can be increased by subsequent heat treatment such as homogenization or aging, as shown in Fig. 1(b,c).

## Conclusions

In conclusion, this study shows that, although the large fraction of grain boundaries in nanostructured materials reduces their plasticity because of the limitations on dislocation activity, the nanostructured materials can exhibit significant plasticity and even room-temperature superplasticity, if their deformation mechanism is tuned to grain-boundary sliding by engineering the grain-boundary segregation and diffusion. This strategy, which we believe is not limited to the Al-Zn alloy examined in this study, introduces a new approach to improving the plasticity of nanostructured materials for structural applications, where high plasticity and deformability is needed at room temperature.

## Electronic supplementary material

Supplementary information
