# Supplementary material for: Transition from poor ductility to room-temperature superplasticity in a nanostructured aluminum alloy
Source: Sci Rep. 2018 Apr 30;8:6740. doi: 10.1038/s41598-018-25140-1 (PMC5928095; doi:10.1038/s41598-018-25140-1)
Supplement: Supplementary file 1 — Supplementary information [file 41598_2018_25140_MOESM1_ESM.doc]

**Supporting Information**

**Transition from poor ductility to room-temperature superplasticity in a nanostructured aluminum alloy**

Kaveh Edalati^1,2,*^, Zenji Horita^1,2^ and Ruslan Z. Valiev^3,4,*^

^1^ WPI, International Institute for Carbon-Neutral Energy Research (WPI-I2CNER), Kyushu University, Fukuoka 819-0395, Japan

^2^ Department of Materials Science and Engineering, Faculty of Engineering, Kyushu University, Fukuoka 819-0395, Japan

^3^ Institute of Physics of Advanced Materials, Ufa State Aviation Technical University, Ufa, Russia

^4^ Laboratory for Mechanics of Bulk Nanomaterials, Saint Petersburg State University, Saint Petersburg, Russia

***Corresponding authors:**

Kaveh Edalati (E-mail: kaveh.edalati@zaiko6.zaiko.kyushu-u.ac.jp)

Ruslan Z. Valiev (E-mail : ruslan.valiev@ugatu.su)

**
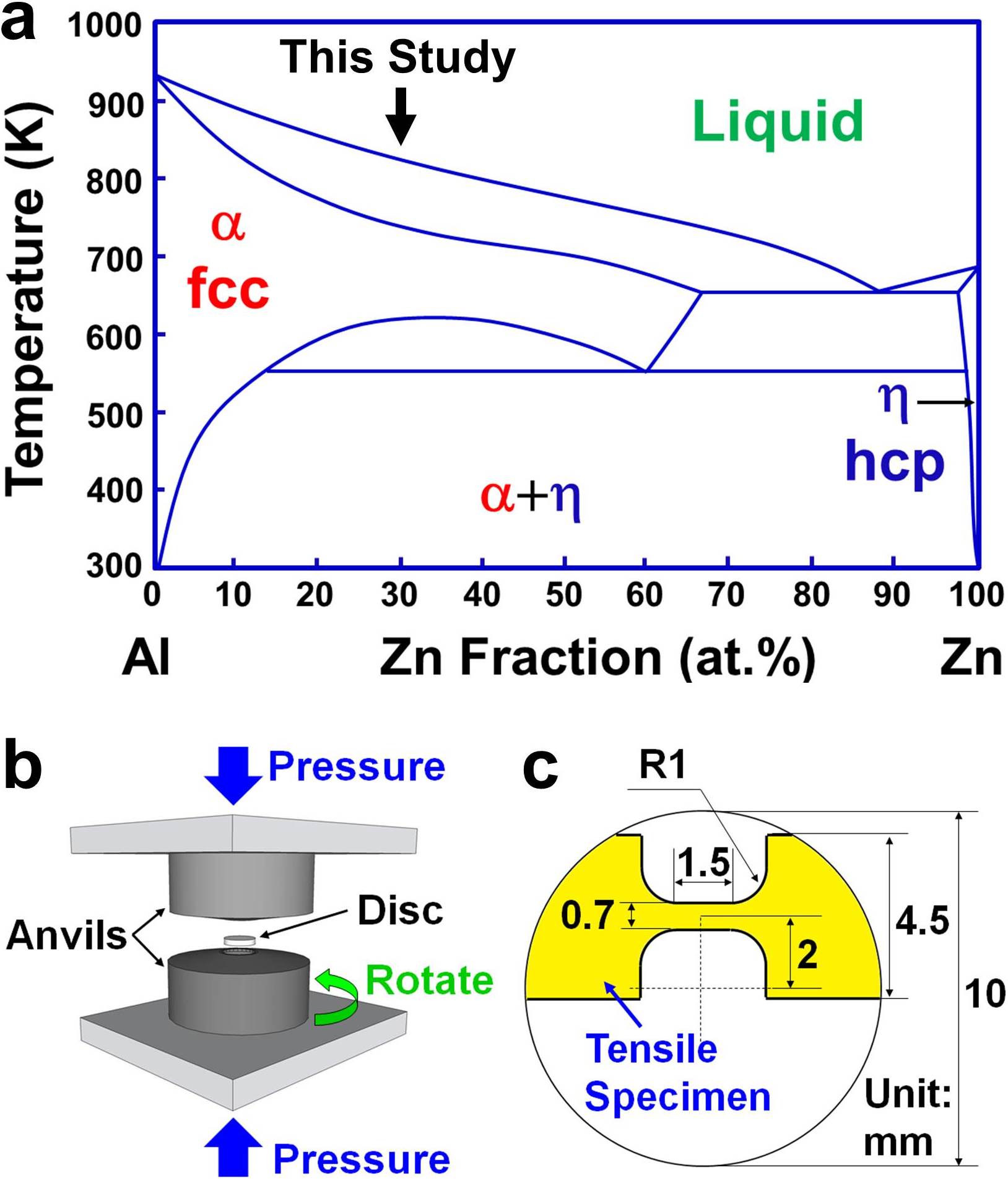
**

**Figure S1.** (a) Phase diagram of the Al-Zn system based on the data reported in Ref. [21]. (b) Schematic illustration of high-pressure torsion which was used as an SPD method in this work. (c) The position and dimensions of tensile specimens prepared from SPD-processed discs.

**
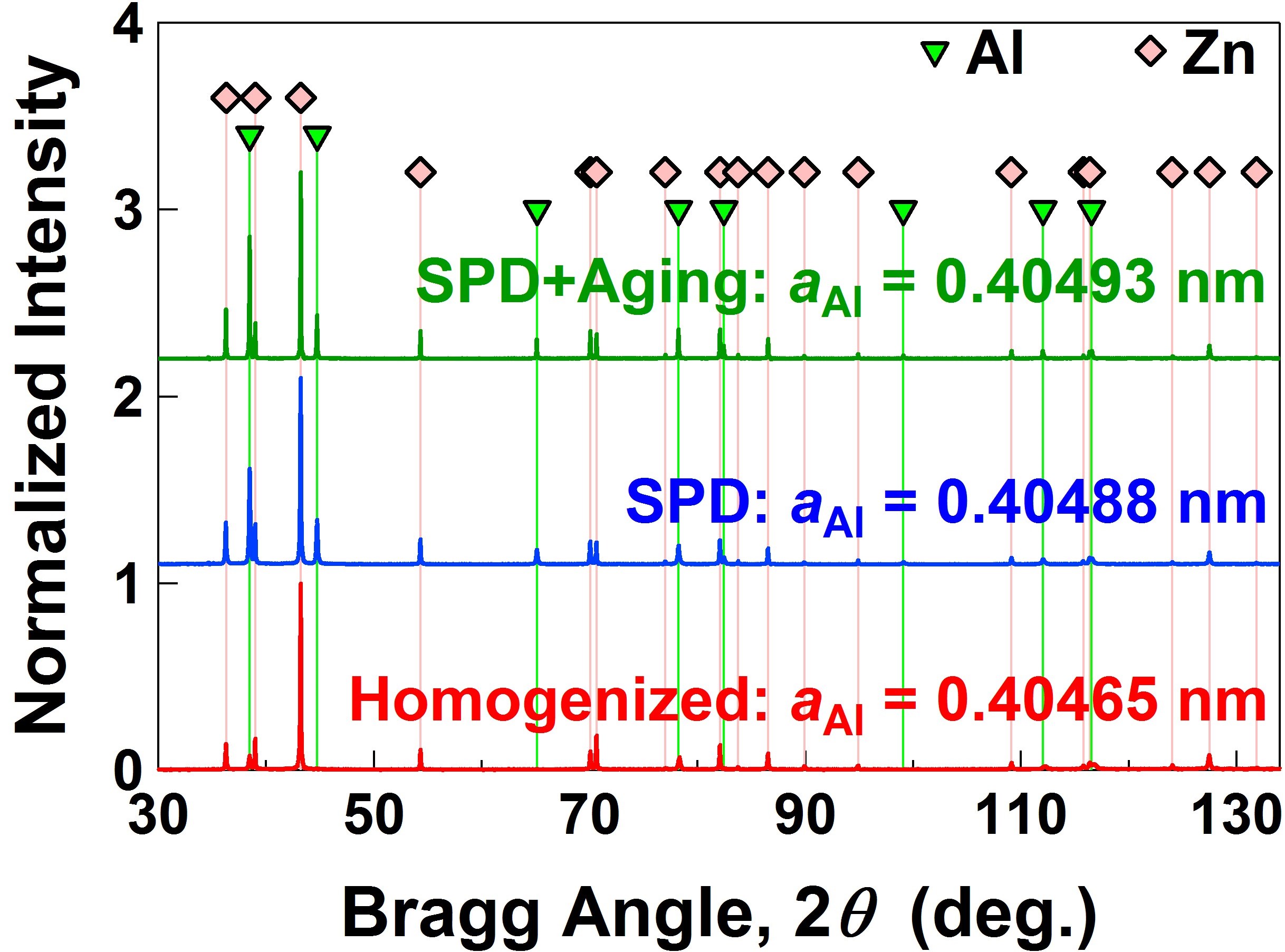
**

**Figure S2.** The Al-Zn alloy contains Al-rich and Zn-rich phases, but the lattice parameter of Al-rich phase (*a*_Al_) increases after SPD and even more after natural aging, indicating the depletion of Al-rich phase from the Zn atoms. XRD profiles of the Al-Zn alloy processed with homogenization, SPD and SPD followed by natural aging for 100 days. The intensities were normalized by the intensity of the most intense peak at a Bragg angle of ~43^o^ for better visibility.

**
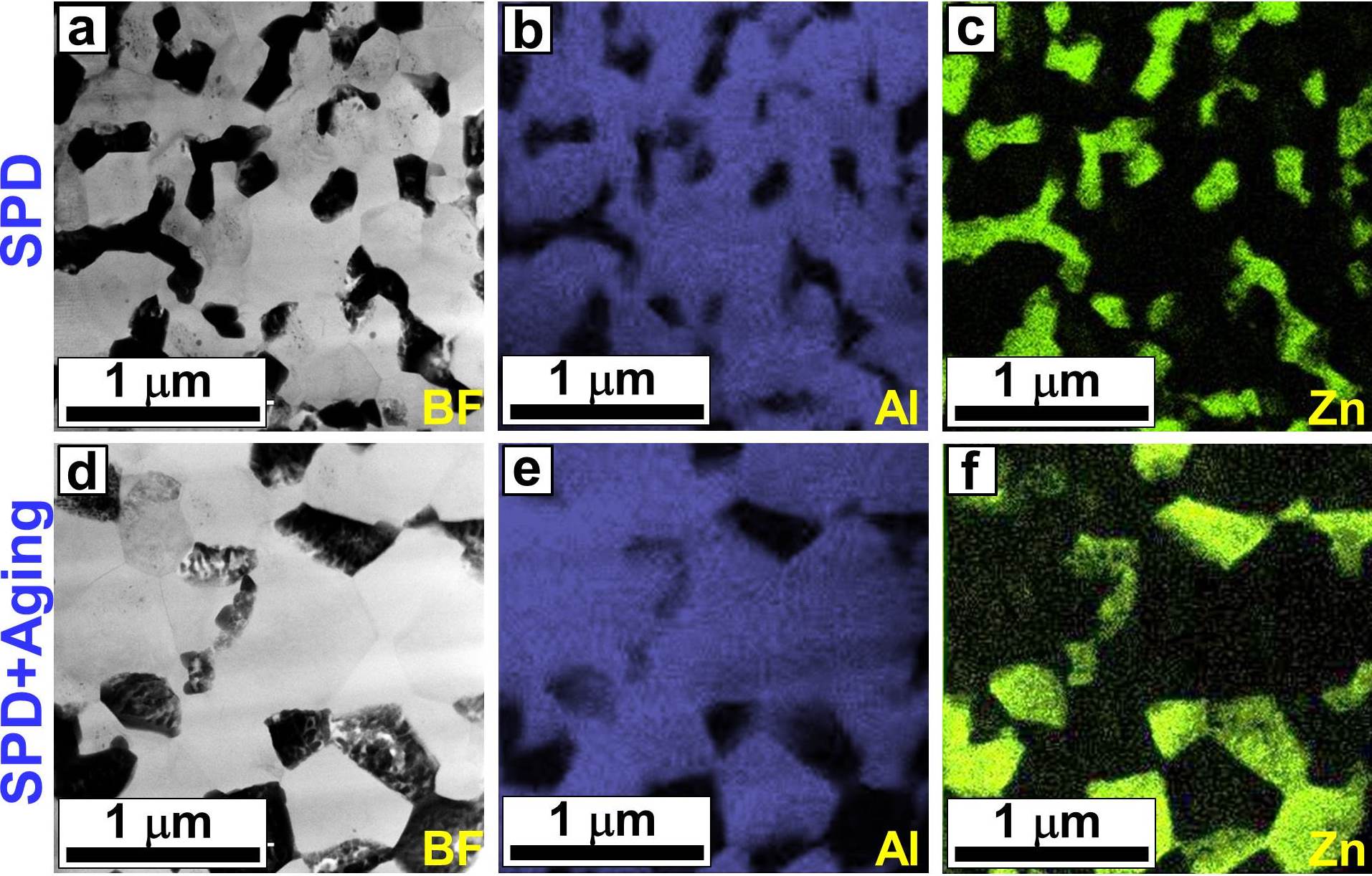
**

**Figure S3.** The average grain size of SPD-processed sample increases after natural aging for 100 days. STEM-BF micrographs and corresponding distribution of Al and Zn examined by EDS in the Al-Zn alloy processed with (a-c) SPD and (d-f) SPD followed by natural aging for 100 days.

**
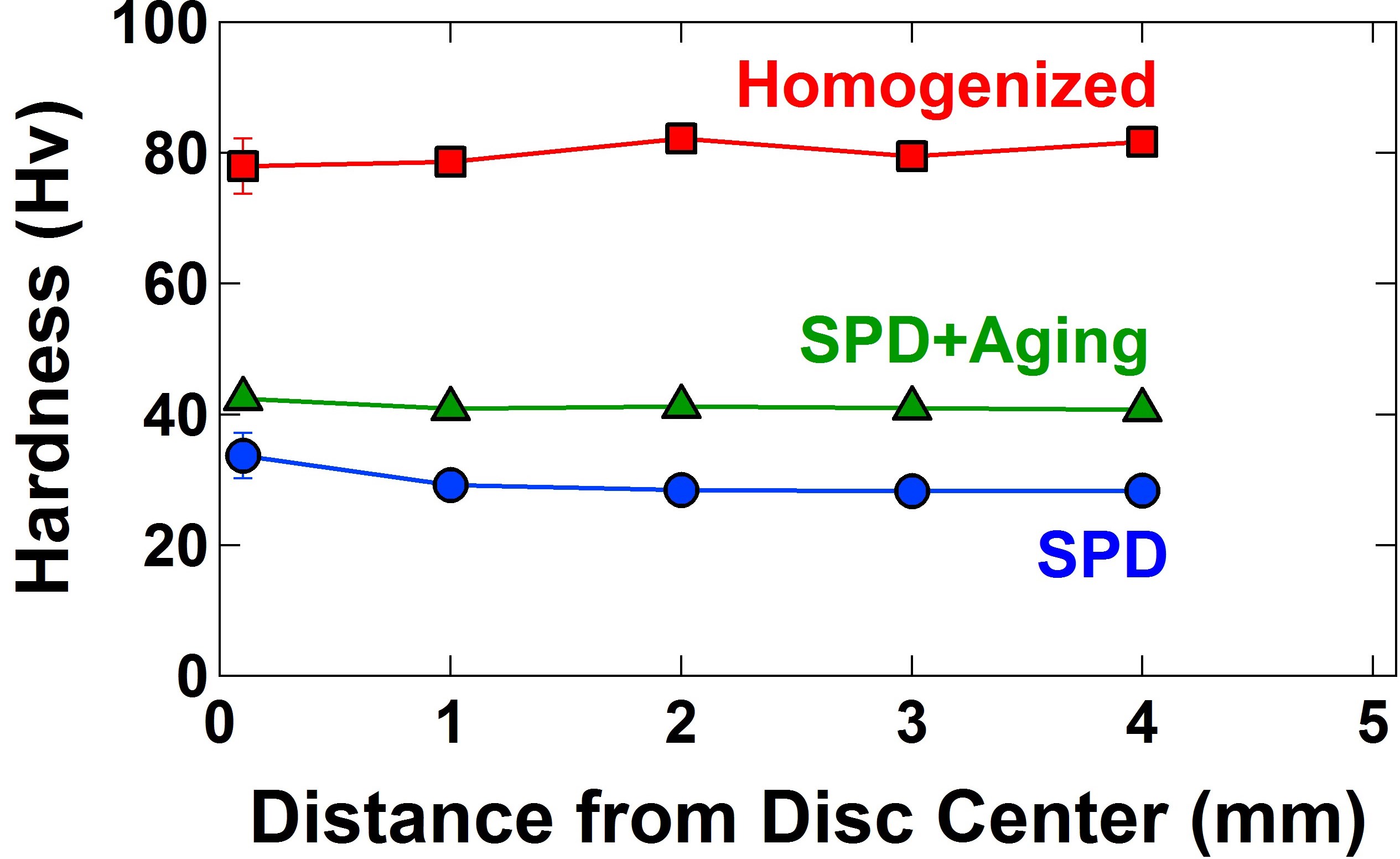
**

**
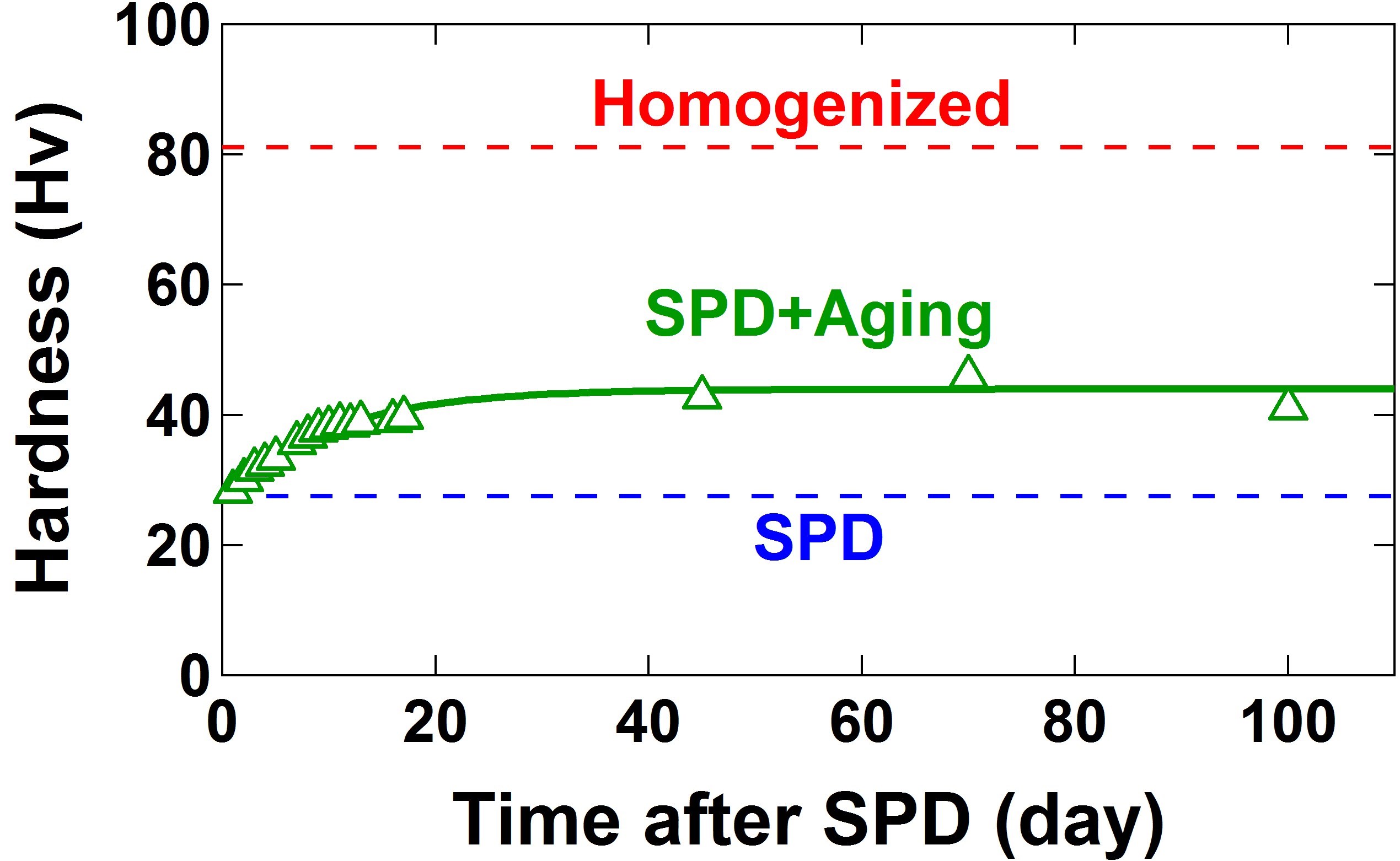
**

**Figure S4.** The hardness decreases after SPD processing but increases after natural aging. (a) Hardness of Al-Zn alloy processed with homogenization, SPD and SPD followed by natural aging for 100 days. (a) Hardness versus distance from the disc center. (b) Hardness against the natural aging time.

**
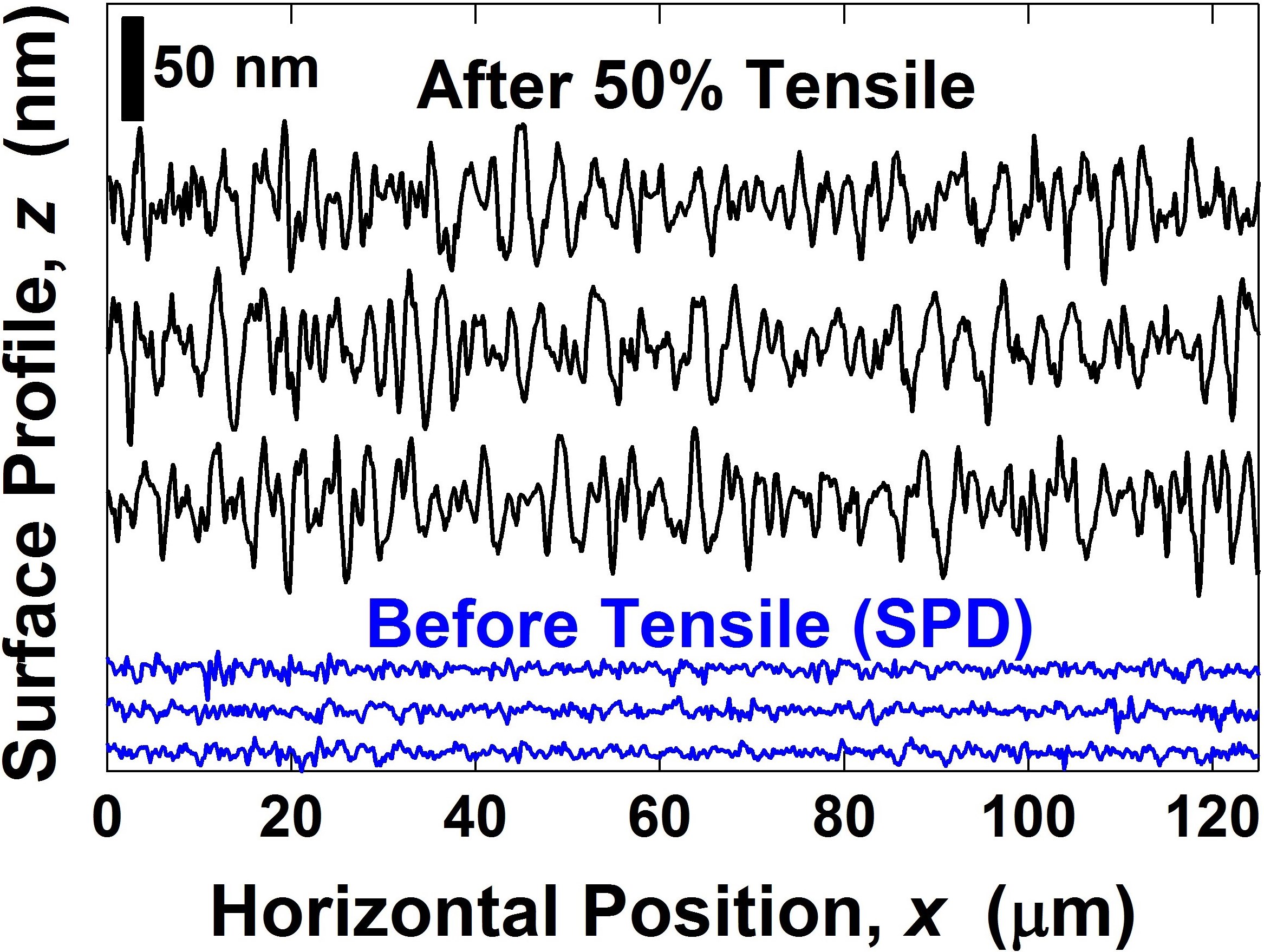
**

**Figure S5.** The surface roughness of the SPD-processed sample increases after pulling the sample for 50%, indicating the occurrence of grain-boundary sliding. Plots of surface profiles on three different areas of sample achieved by laser microscopy before and after tensile test for 50% elongation under a strain rate of 5.5×10^-5^ s^-1^ for the sample processed by SPD.

**
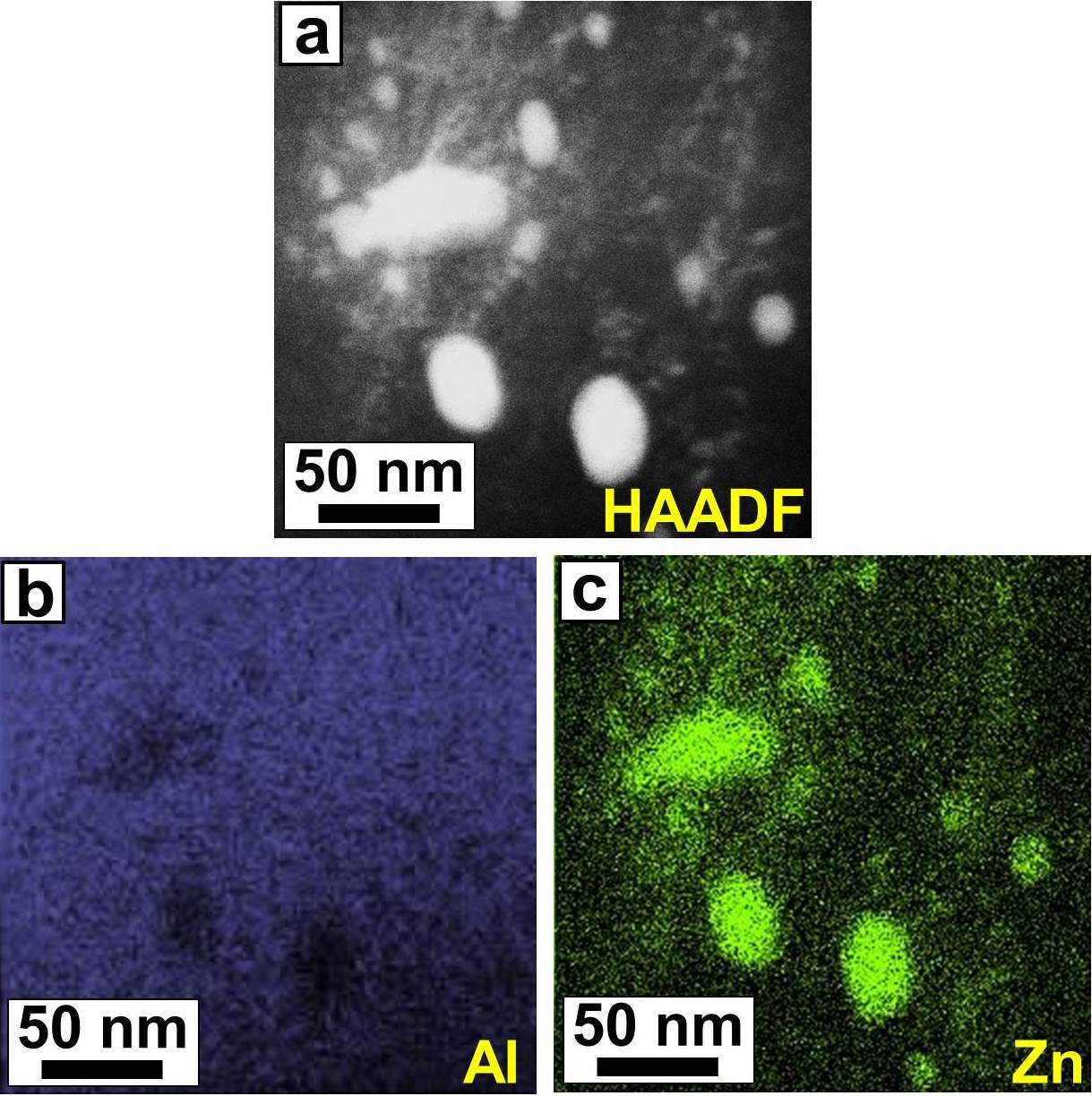
**

**Figure S6.** Zn precipitates form after natural aging for 100 days. HAADF image and corresponding EDS mapping for distribution of Al and Zn in the Al-Zn alloy processed with SPD followed by natural aging for 100 days.
